# HIV testing and counselling for migrant populations living in high-income countries: a systematic review

**DOI:** 10.1093/eurpub/cks130

**Published:** 2012-09-23

**Authors:** Debora Alvarez-del Arco, Susana Monge, Amaya Azcoaga, Isabel Rio, Victoria Hernando, Cristina Gonzalez, Belen Alejos, Ana Maria Caro, Santiago Perez-Cachafeiro, Oriana Ramirez-Rubio, Francisco Bolumar, Teymur Noori, Julia Del Amo

**Affiliations:** 1 National Centre of Epidemiology, Instituto de Salud Carlos III, Madrid, Spain; 2 Department of Health Sciences, the Universidad Rey Juan Carlos I, Madrid, Spain; 3 Division of Environmental and Reproductive Epidemiology, Spanish Network for Research in Epidemiology and Public Health (Biomedical Research Centre Network for Epidemiology and Public Health [CIBER de Epidemiología y Salud Pública] CIBERESP), Spain; 4 Department of Public Health Sciences, Faculty of Medicine, University of Alcalá, Madrid, Spain; 5 Public Health Capacity and Communication Unit, European Centre for Disease Prevention and Control (ECDC), Stockholm, Sweden

## Abstract

**Background:** The barriers to HIV testing and counselling that migrants encounter can jeopardize proactive HIV testing that relies on the fact that HIV testing must be linked to care. We analyse available evidence on HIV testing and counselling strategies targeting migrants and ethnic minorities in high-income countries. **Methods:** Systematic literature review of the five main databases of articles in English from Europe, North America and Australia between 2005 and 2009. **Results:** Of 1034 abstracts, 37 articles were selected. Migrants, mainly from HIV-endemic countries, are at risk of HIV infection and its consequences. The HIV prevalence among migrants is higher than the general population’s, and migrants have higher frequency of delayed HIV diagnosis. For migrants from countries with low HIV prevalence and for ethnic minorities, socio-economic vulnerability puts them at risk of acquiring HIV. Migrants have specific legal and administrative impediments to accessing HIV testing—in some countries, undocumented migrants are not entitled to health care—as well as cultural and linguistic barriers, racism and xenophobia. Migrants and ethnic minorities fear stigma from their communities, yet community acceptance is key for well-being. **Conclusions:** Migrants and ethnic minorities should be offered HIV testing, but the barriers highlighted in this review may deter programs from achieving the final goal, which is linking migrants and ethnic minorities to HIV clinical care under the public health perspective.

## Introduction

Migrant populations, largely from sub-Saharan Africa (SSA), represent a considerable proportion of HIV infections in Europe and are heavily affected by late HIV diagnoses.^1^^,^^2^ A similar situation occurs in ethnic minorities in the USA.^3^ In 2006, the US Centers for Disease Control and Prevention published their revised recommendations for HIV testing, which aimed for routine voluntary HIV screening for all persons aged 13–64 years in health-care settings independently of HIV risk assessment unless the local HIV prevalence falls below 0.1%. Under the new recommendations, neither pretest counselling nor separate signed consent forms are required.^3^ Since then, other recommendations have been issued to expand HIV testing and normalize testing.^4–8^ Although increasing HIV testing at population level is a challenge, doing so in migrants and ethnic minorities may pose additional difficulties. Whereas some barriers to HIV testing are shared by other groups, others, such as administrative,^9–12^ legal,^1^^,^^13^^,^^14^ language^14–18^ and cultural barriers,^9^^,^^11^^,^^14^^,^^16^^,^^18–20^ are not. Some European Union (EU) countries do not provide HIV care for people of uncertain legal status. Therefore, the lack of any medical benefit for individuals found to have HIV combined with the fear of deportation may hinder public health initiatives promoting testing in migrants.^21^

The different epidemiological patterns of HIV infection in migrants and ethnic minorities in Europe and the migrants’ unique barriers to HIV testing and care may compromise proactive HIV testing approaches that rely on the statement that HIV testing is linked to care.^4^^,^^5^^,^^22^ Given that the translation of policies into practice requires deep knowledge of target populations, we have conducted a systematic review of the literature on HIV testing and counselling targeting migrants living in high-income countries.

## Methods

We performed a literature search in PubMed, EMBASE, CRD York database, Cochrane and Web of Knowledge of articles published in English between 2005 and 2009 in Europe, North America and Australia. To be eligible, articles had to include at least one keyword from: (i) ethnic groups, minority groups, transients and immigrants, refugees; (ii) mass screening, diagnosis, AIDS Serodiagnosis, public policy, prevention and control, Centers for Disease Control and Prevention (USA), counselling; and (iii) Acquired immunodeficiency syndrome, HIV, HIV Infections.

After piloting in 14 articles, the final data collection form included information on year and country of the study, ‘migrants’ and ‘ethnic minorities’ definition, setting, study design and sample size. Information on gender, criminalization of HIV transmission and legal consequences of HIV disclosure in migrants with uncertain status was also collected.

**Figure 1:**
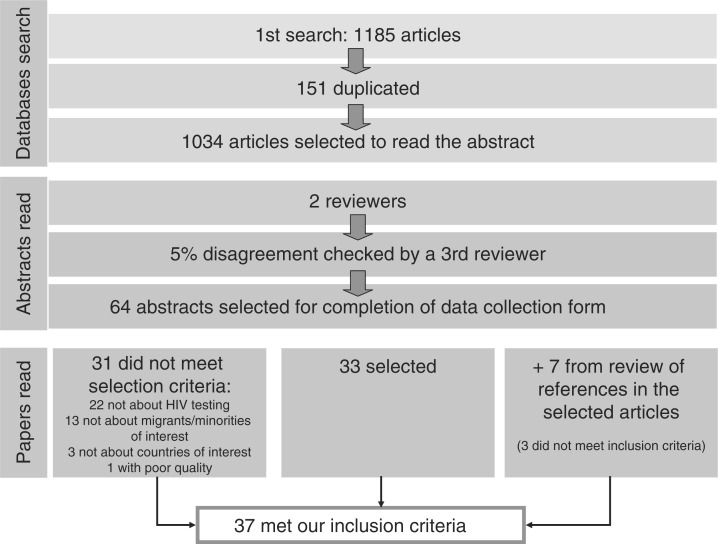
Flow diagram

As described in figure 1, 1185 articles were identified; 151 were duplicates. The remaining 1034 abstracts were read, and only abstracts not referring to migrants or ethnic minorities in Europe, USA, Canada and Australia and HIV testing and/or counselling were eliminated at this stage; 970 articles were discarded and 64 selected for full review.

Teams of two people read the 64 articles and applied eligibility criteria; 31 articles were rejected because they did not deal with HIV testing (*n* = 22), they did not deal with migrants or ethnic minorities (*n* = 13), countries did not meet criteria (*n* = 3) or they were of poor quality (*n* = 1). The review of these 64 articles identified seven new manuscripts, from the manuscripts’ references, of which four met inclusion criteria. Final analyses were based on 37 articles.

## Results

Most studies were conducted in Anglo-Saxon countries—12 in USA, 4 in Canada, 13 in the UK—and 8 from other European countries. None of the articles was from Australia. The majority (*n* = 35) was conducted in one country, most (*n* = 20) with local scope. Studies were conducted in health (*n* = 15) and community settings (*n* = 11) (supplementary table). Overall, 25 studies (68%) used quantitative methodology, two combined quantitative and qualitative methodology, six used qualitative methodology and four were literature reviews. Operational definitions for ‘migrant’, ‘race’ or ‘ethnicity’ (understood as concept defining racial/ethnic minorities) were present in all articles; more than one definition was often present; 14 defined migrants based on country of origin, 13 on country of birth and 3 on nationality. With regards to race and ethnicity, eight studies (mostly from USA) defined subjects on self-reported ethnicity and nine on documented ethnicity (*n* = 7), ethnic origin (*n* = 1) and race (*n* = 1).

Migrant populations studied were largely sub-Saharan Africans,^10^^,^^11^^,^^23–26^ Latin Americans^23^ and South Asians^20^ in EU countries, and Hispanics or Latinos,^12^^,^^15^^,^^17^^,^^27–29^ Africans^16^ and Southeast Asians^14^ in the USA. Three articles from Canada studied asylum seekers, visa applicants and refugees^9^^,^^30^^,^^31^, and one from the USA studied adopted foreign children.^19^ The racial/ethnic minorities were largely ‘Black communities’ in the UK.^32–35^ One study in the USA refers to generic ‘racial minorities’.^36^

The 37 studies addressed heterogeneous aspects of HIV testing and counselling in migrants and ethnic minorities, which could be grouped as follows: (i) prevalence and risk factors for HIV infection; (ii) barriers to HIV testing; (iii) HIV testing uptake; (iv) late HIV diagnosis; and (v) interventions to encourage HIV testing.

### Prevalence and risk factors for HIV infection in migrants and ethnic minorities

Although our aim was not to systematically review HIV prevalence and risk factors in migrants and ethnic minorities, this topic came up frequently. Although many articles include general references to this issue, studies that report data about differential prevalence of HIV have been included in table 1. The groups identified as having high HIV prevalence were sub-Saharan Africans in Europe, black Africans and black Caribbeans in London and sub-Saharan Africans, Latinos and blacks in USA.

**Table 1:** HIV prevalence in migrants and ethnic minorities

Authors	Country/Date	Sample size	Target population	HIV prevalence/incidence	Design
Monge-Maillo B et al	Spain (2009)	2198	Immigrants referred to the Tropical Medicine Unit of Ramón y Cajal Hospital (Madrid) over a 20-year period	Incidence: total population: 97 (4.4%); sub-Saharan Africans: 82 (5.2%); Latin Americans: 15 (2.4%)	Cross-sectional study
Forbes KM et al.	UK (2008)	30	Patients attending an outreach clinic for those <25 years between June and October 2007, in an area where black and minority ethnic groups comprise the majority of the local population	There were no cases of HIV	A retrospective case-notes review was undertaken of those attending community-based sexual health services. (Note: not clear if all respondents were from minorities)
Zencovich M et al.	Canada (2006)	634 958	All applicants in Canada, 15 years of age and older, for permanent residency between 2002 and 2003	Incidence: 932 (0.146%). Prevalence of 3% among applicants from six African countries (Zimbabwe, Burundi, Rwanda, Uganda, Zambia and Chad)	National data collected by Public Health Agency of Canada
Schmid J et al.	Denmark (2005)	Does not know (Unknown) (DK)	Children <16 years in Denmark in 2003	Incidence: 89 (5.77 per 100 000 children). Of these 89 newly diagnosed: 48% born in Denmark 43% in Africa and 9% in other places	Data from the national surveillance system and HIV-infected children from the Danish Paediatric HIV Cohort Study
MacPherson DW et al.	Canada (2006)	256 970	Residency applicants <15 years of age between 2002 and 2005	36 (0.014%), most of them from Africa (89%)	Data from the Canadian immigration medical examination register
Perez-Molina JA et al.	Spain (2009)	1609	Immigrants referred to the Tropical Medicine Unit of Ramón y Cajal Hospital (Madrid) during 1997–2006	77 (4.8%). By geographic groups: sub-Saharan Africans (5.6%); South-Central Americans (3.2%)	Non-interventional retrospective medical chart review
Dougan, S et al.	UK (2005)	1040	Men who have sex with men aged 16–44 years in England and Wales in 2002	Prevalence: 7.4% black and minority ethnic MSM; 3.2% white MSM	Cross-sectional study with the data from the Survey of Prevalent HIV Infections Diagnosed that estimates the number of individuals living with diagnosed HIV infection in England and Wales (E&W) since 1995

Epidemiological patterns in countries of origin, together with clinical presentations in countries of destination, led some authors to conclude most HIV infections in migrants are imported. This approach is largely applied to migrants from SSA in Europe (Denmark,^37^ France,^38^ Spain^18^^,^^23^ and UK^25^^,^^32^^,^^34^) and Canada.^9^^,^^30^^,^^31^ Other studies consider some migrants may have acquired HIV in countries of destination because of unfavourable environment to fostering preventive behaviours.^11^^,^^39^^,^^40^ Some migrants are described as having high-risk behaviours: multiple sexual partners, low and inconsistent condom use, high alcohol consumption and drug use.^17^^,^^27–29^

Migrant women and ethnic minority men who have sex with men (MSM) are considered especially vulnerable; female migrants suffer disproportionate burden of HIV infection derived from difficulties negotiating condom use^28^ and sexual exploitation.^31^ The invisibility of ethnic minority MSM, who go unnoticed by the stigma of being MSM that would involve their country or ethnic group of origin, is thought to be key to their vulnerability to HIV infection; they have high HIV prevalence, unsafe sex^39^ and high prevalence of late HIV diagnosis.^14^^,^^35^

### Barriers to HIV testing in migrants and ethnic minorities

Barriers to HIV testing exist at the structural, health-care provider, health-care user and community levels.

Structural barriers to HIV testing relate to poor living conditions, legal and administrative status and discrimination in countries of destination. High levels of unemployment and poverty in migrants and ethnic minorities, low social status and inequalities are mentioned as barriers to HIV testing and care.^10^^,^^11^^,^^18–20^^,^^24^ Legal status, which ranks among the highest priorities, and concerns about implications of testing positive are the main barriers in studies from Canada, USA, Spain and the UK. According to these studies, in contexts where an HIV diagnosis may adversely affect visa or residence application or where there is a fear of deportation, migrants are reluctant to be tested.^10^^,^^11^^,^^13^ Lack of entitlement to health care for undocumented immigrants is another barrier, largely but not exclusively, mentioned in the USA.^13^^,^^14^^,^^35^ Lack of clarity among health-care providers on migrants’ rights to health care is highlighted.^24^ Regarding stigma and discrimination, African people tested for HIV in London in the 1990s were twice as likely as whites to be concerned about being discriminated against.^10^ Structural racism is also mentioned by Fakoya et al.,^24^ who drew attention to the disproportionate number of sub-Saharan Africans prosecuted for allegedly transmitting HIV infection in the UK.

Within health-care structures, communication, language problems and lack of cultural sensitivity and underinvestment in culturally competent services lead to misconceptions about migrants and minority groups by health-care providers.^14–18^^,^^20^ Some migrants and ethnic minorities prefer to visit medical practitioners from their own community.^19^ Whereas some communities are described as having good knowledge about where to go for HIV testing, for example, sub-Saharan Africans in the UK,^11^ other studies identify poor knowledge among migrant and ethnic minorities regarding where to get tested for HIV anonymously and free of charge.^9–12^ Other barriers at health-care user level include the low-priority migrants assigned to health care^11^^,^^13^ and their low perception of HIV risk^10^^,^^12^^,^^14^^,^^35^^,^^41^. Migrants give priority to basic needs, but knowledge of their HIV status ranks low.^13^ Low HIV risk perception at individual level is mentioned, even in people with community risk awareness.^10^^,^^12^^,^^14^^,^^35^^,^^41^ Researchers note there is often a gap between risk perception and individual risk behaviours^14^; some studies describe low risk perception among those who test HIV positive.^10^^,^^11^^,^^41^

At the community level, cultural and gender norms may dissuade heterosexual men and women and MSM from seeking HIV testing. Loss of status and community support and social isolation after confidentiality gaps are among the main reasons given for not testing.^9–13^^,^^16^^,^^24^^,^^26^^,^^27^^,^^34^^,^^41^^,^^42^ This is a particular concern for individuals who need to bring a relative or friend to help with translation.^19^ Olshefsky et al.^27^ report ‘machismo’ as a barrier among Latino men; Foley^16^ reports that women from SSA have difficulty seeking HIV testing or treatment without partners’ approval and economic support.

### HIV testing uptake in migrants and ethnic minorities

Several studies describe an HIV testing uptake in migrants and ethnic minorities to range from 21% to 73% in the USA and from 23% to 64% in Europe^12^^,^^14^^,^^20^^,^^29^^,^^43^ (table 2). Overall, a higher proportion of ethnic minority and migrant women have been tested for HIV compared with men; this is partially owing to women’s acceptance of routine HIV screening during antenatal care.^29^^,^^33^^,^^42^^,^^44^ However, beyond this, several studies support a gender difference in HIV testing uptake, with migrant men being not only less exposed to HIV testing but also less willing to be tested.^24^

**Table 2:** Uptake of HIV test in migrants and ethnic minorities

Authors	Country/Date	Sample size	Target population	Test prevalence/test acceptance prevalence	Design
Forbes KM et al.	UK (2008)	117	Outreach clinic for those <25 years in an area where black and minority ethnic groups comprise the majority of the local population	23% ever tested	A retrospective case-notes review was undertaken of those attending community-based sexual health services. (Note: not clear whether all respondents were from minorities)
Conaty SJ et al.	UK (2005)	443	Sub-Saharan women in antenatal care	86% accepted an HIV test	Cross-sectional study. HIV test acceptance. (Note: prevalence calculated by our research team based on presented article data)
Fernandez MI et al.	USA (2005)	244	Hispanic migrant/seasonal farm workers in southern Miami-Dade County, Florida	21% (51/244) had been tested for HIV; 39% (94/244) declared they would accept on the day of the interview; 69% (134/193 never tested) declared they would accept if recommended by a provider	Cross-sectional study: questions about HIV test performance and intention to test
Sadler KE et al.	UK (2006)	114	Black Africans (>16 years old) living in London	82% (93/114) accepted HIV testing in the survey	Cross-sectional study with offer of HIV test
Dowling T et al	USA (2007)	627	Participants at black gay, Hispanic gay or gay pride events	24% (133) of those with unknown or negative HIV status (543) accepted HIV testing	Cross-sectional study with offer of HIV test. (Note: not clear if all respondents were from ethnic minorities. Of all persons willing to be tested, not all were finally tested for several reasons, mainly resource limitations)
Ostermann J et al.	USA (2007)	146 868	Adult participants in the survey aged 18–64 years	Tested in past 12 months (by ethnicity): white non-Hispanic, 8.1%; black non-Hispanic, 19.0%; Hispanic, 11.7%; Other, 9.6%. Plan to test in next 12 months: white non-Hispanic, 5.2%; black non-Hispanic, 19.8%; Hispanic, 12.7%; Other, 7.1%	Cross-sectional analysis of data from 146 868 participants aged 18–64 years in the 2000–05 National Health Interview Surveys, HIV test in the past
Tariq S et al.	UK (2007)	458	Cases were defined as the first 125 new Genito-Urinary (GU) clinic attendees who self-identified as South Asian. Controls were defined as subsequent new presentations self-identified as non-South Asian	Ever tested: cases: 60% (148/229); controls: 64% (154/229)	A retrospective case–control study was performed at a GU Clinic in London: HIV test in the past
Huang ZJ et al.	USA (2008)	604	Self-identified as Cambodian, Laotian or Vietnamese; >18 yearsold and residents in Washington, DC	Ever tested: total sample, 31% (186/604); Laotians, 22% (44/196); Vietnamese, 37% (72/197); Cambodians, 38% (79/211)	Cross-sectional study. Have had an HIV test
Southgate J et al.	UK (2008)	1586	Pregnant women from ethnic minority groups	Prevalence ratio of HIV test acceptance: white, 91% (1094/1214); black African, 92% (145/158); Asian, 90% (138/153); Chinese, 80% (4/5)	Cross-sectional study. Antenatal HIV screening routinely proposed
López Quintero C et al.	USA (2005)	4261	Hispanic subgroups living in the USA	Ever tested: total sample, 34% (1444/4261); Puerto Ricans, 44% (197/444); Mexicans, 28% (419/1480); Mexican Americans, 33% (355/1079); Cubans + Cuban Americans, 29% (80/277); Central/South Americans, 41% (259/640); other Hispanics, 39% (133/341)	Cross-sectional study. Have had an HIV test (Note: prevalence by our research team based on presented article data)
Dougan S et al.	UK (2005)	1040	Black and minority ethnic men who have sex with men in England and Wales	Prevalence ratio of HIV test acceptance: Caribbean, 52% (138/265); Central/South America, 60% (593/993); sub-Saharan African, 54% (473/870); Asia, 56% (417/739)	Cross-sectional study with offer of HIV test

The context in which testing is offered and who offers the test seem to be important determinants of test acceptance. For example, in a US study, 70% of Hispanic migrant farm workers reported they would accept testing recommended by a health-care provider, with women more likely than men to accept testing.^29^ In contrast, MSM from ethnic minorities in the USA were more prone to accept HIV testing outside health settings; rapid HIV testing of MSM in racial/ethnic minority groups in settings such as gay pride events is a useful way to enable HIV-infected MSM to learn their HIV status.

### Late HIV diagnosis in migrants and ethnic minorities

High prevalence of delayed diagnosis of HIV infection in migrants and ethnic minorities was reported, largely among sub-Saharan Africans in the UK,^10^^,^^11^^,^^13^^,^^24^^,^^25^^,^^40^ Spain^23^ and France^38^ and among Latinos and Asian Americans in the USA^14^^,^^27^ and Canada.^45^

López-Quintero et al.^12^ report Hispanics to be more likely to have delayed HIV diagnoses than whites and Afro-Americans. Prost et al.^10^ cite the Health Protection Agency and the Mayisha II Study,^46^ which estimated that by 2005, approximately 21 500 Africans were living with HIV in the UK and that one-third of them were undiagnosed. Fakoya et al.^24^ report that most people from sub-Sahara diagnosed with HIV in the UK acquired the infection in the countries of origin and tend to present with advanced disease. Also in the UK, Chadborn et al.^13^ report high prevalence of late diagnosis in heterosexual whites (36%), black Caribbeans (36%) and black Africans (43%). They describe lower rates of delayed diagnosis in women diagnosed through antenatal testing than other people diagnosed elsewhere. For sub-Saharan Africans, late diagnosis was present in 21% of women diagnosed during antenatal care, 44% of women diagnosed elsewhere and 50% of men. In France, although women were less likely to be diagnosed late because of routine prenatal testing, this was not the case among migrants. Acquisition of HIV infection at early ages before arrival to France could underlie these differences.^38^

### Interventions to encourage HIV testing in migrants and ethnic minorities

The literature identified two main approaches to HIV testing: general population approaches in health-care settings and targeted approaches directed to at-risk populations, including migrant and/or ethnic minorities. In both cases, specific interventions to encourage the participation of migrants and ethnic minorities are discussed. Among the targeted approaches, voluntary or compulsory HIV testing strategies are analysed.

Antenatal screening is an example of a population-wide approach; various studies support routine antenatal testing for all pregnant women as an effective strategy for achieving good coverage of HIV testing in migrant and ethnic minorities.^42^ Regarding preferences of immigrant women, a study in Canada found that women from HIV-endemic countries prefer non-targeted strategies integrated within health services.^9^ Some countries have taken targeted approaches to HIV testing in antenatal care. For example, Denmark switched from routine to HIV selective antenatal screening in 1995; HIV testing was only offered to women from high prevalence countries since almost all HIV-positive pregnant women identified were migrants or married to migrants from high prevalence countries.^37^ However, a few HIV-positive babies were born subsequently, resulting in the reintroduction of universal screening.

Health-care provider endorsement was identified as a significant predictor of HIV testing. Some authors recommend mixed approaches; introducing HIV screening in routine medical practice in addition to targeted strategies in place, particularly in areas with high HIV prevalence and a concentration of migrant population.^9^^,^^29^^,^^41^^,^^42^

Many authors indicated that culturally sensitive HIV testing and counselling interventions are needed to improve institutional access to health services to promote HIV testing among migrants and ethnic minorities.^9^^,^^11^^,^^29^ Community partnerships and participation are identified as critical to increase testing uptake. A UK study calls for community involvement in promoting testing along with the benefits of accessing antiretroviral therapy.^11^ Provision of testing services in client’s language is also mentioned.^9^^,^^17^

Innovative targeted approaches to reach people who might not otherwise use testing services include the provision of HIV rapid testing in non-traditional health-care settings outside normal working hours.^29^ Provision of point-of-care testing by non-government and community-based organizations and testing by outreach services, mobile clinics and in venues such as barber shops and hair salons, social clubs, sporting events or street corners^27^^,^^36^ are recommended. Offering HIV testing at gay pride events^36^, saunas^41^ and other venues was also considered to increase uptake among MSM.

Another approach specifically targeting migrants is screening on arrival in the country. US policy requires HIV testing for asylum seekers,^47^ and in 2008, when the mentioned article was published, those who were HIV-infected could only obtain temporary admission to the USA. Children who arrive in the USA needing a permanent visa are required to be screened for HIV, hepatitis B and C and tuberculosis, and screening is repeated 6 months after arrival. The implications of HIV test results for visa status are not discussed.^19^ HIV testing is also mandatory for migrants and asylum seekers entering Canada. Screening includes clinical referral and information that is sensitive to the gender, cultural and linguistic profile of the client.^30^^,^^31^ This approach is reported to have resulted in an increased number of cases of HIV diagnosed in immigrants and uptake of health care in this country.^45^ A French study recommends offering HIV testing to migrants on arrival to reduce late diagnosis.^38^

## Discussion

This systematic review has found that migrants, largely those from HIV-endemic countries, are at high risk of HIV infection and its consequences.^10^^,^^11^^,^^13^^,^^14^^,^^23–25^^,^^27^^,^^38^^,^^40^^,^^45^ Their HIV prevalence is higher than that of the general population, they have a higher frequency of delayed HIV diagnosis and are more vulnerable to the negative effects of disclosure of HIV status.^11^^,^^16^ For migrants from countries where HIV prevalence is low, their socio-economic vulnerability puts them at risk of acquiring HIV in destination countries.^11^^,^^39^^,^^40^ In addition to their socio-economic vulnerability and the barriers to HIV testing shared with other groups at risk, migrants have specific legal and administrative impediments to accessing health services^10^^,^^16^^,^^17^^,^^24^ and thus HIV testing facilities—in some countries, undocumented migrants are not entitled to health care^13^^,^^14^^,^^35^—as well as cultural and linguistic barriers,^14–18^^,^^20^ racism and xenophobia^10^; criminalization of HIV transmission has disproportionately affected migrants from SSA.^10^ Migrants also fear stigma and discrimination from their communities, fundamental for their well-being in a foreign country.^10^

Testing migrants for HIV may take place either in the context of general population strategies in health-care settings—such as antenatal testing—or targeted approaches among specific populations. HIV testing uptake in antenatal settings in migrants is high and similar to that of non-migrant women. Women from HIV-endemic countries prefer general population strategies that are integrated within health services.^33^^,^^42^ Overall, a higher proportion of migrant women have been tested for HIV compared with men, partially because of high acceptance of routine antenatal HIV screening, but men, although less exposed to HIV testing, are also less willing to be tested.^29^ Reaching migrant men, both heterosexual and MSM, is a challenge. The literature shows that migrant MSM are a hard-to-reach group and that ‘machismo’ and homophobia are deterrents to HIV prevention.^27^ Mandatory HIV testing for migrants and asylum seekers when entering a country, in place in some settings,^30^^,^^31^^,^^47^ violates the core principles that HIV testing must be confidential, voluntary and performed with informed consent.

A key aspect—often undermined—is the heterogeneity of migrant populations, which requires tailored strategies for some groups. It is essential to discuss and implement interventions in partnerships with the communities. Test performance in non-traditional settings^27^^,^^36^^,^^41^ and during off-hours,^29^ the use of rapid tests^12^^,^^29^^,^^36^ and providing test services from a culturally sensitive perspective appear to be positive interventions. It is remarkable that little information on counselling was retrieved from the literature.

There are a number of study limitations that merit discussion. Although our aim was not to review studies on HIV prevalence and risk factors in migrants and ethnic minorities, these subjects came up in a large number of studies. However, we realize that the search strategy used provides only a partial view on these topics. We are also aware this search covers only articles in English; research in other languages, the grey literature and conference abstracts are not included. The methodology used in this systematic review does not permit a comprehensive assessment of the extent to which mandatory HIV testing is applied to migrants.

There is increasing evidence in recent years of the benefits of HIV testing at the individual and community levels.^5^^,^^48–52^ There is also overwhelming consensus that HIV testing cannot be the final goal, and it is essential to link testing with care, support and treatment so as to ensure a referral pathway for those who test positive. In December 2010, the European Centre for Disease Prevention and Control (ECDC) launched ‘HIV testing: increasing uptake and effectiveness in the European Union’^8^ in which migrants receive considerable attention. The results from this systematic review may help to design interventions targeting migrants, so that ECDC guidance may influence national recommendations.

## Supplementary Data

Supplementary Data are available at *EURPUB* online.

## Funding

This work has been financed by the European Centre for Disease Prevention and Control, the Spanish Network of HIV/AIDS Research [RIS—RD06/0006] and the CIBERESP (Biomedical Research Centre Network for Epidemiology and Public Health).

*Conflict of interest*: None declared.

**Key points**

- Migrants, mainly from HIV-endemic countries, are at high risk of HIV infection and its consequences. Their HIV prevalence is higher than in the general population, they have higher frequency of delayed HIV diagnosis and are more vulnerable to the negative effects of disclosure of HIV status. For migrants from countries with low HIV prevalence, socio-economic vulnerability puts them at risk of acquiring HIV in destination countries.
- Migrants have specific legal and administrative impediments in accessing health services and HIV testing—in some countries, undocumented migrants are not entitled to health care—as well as cultural and linguistic barriers, racism and xenophobia. Migrants fear stigma from their communities, yet community acceptance is key for well-being.
- Although there is increasing evidence of the benefits of HIV testing at both the individual and community levels, the barriers highlighted in this systematic review may prevent programs from achieving the final goal, which is linking migrants and ethnic minorities to HIV clinical care under the public health perspective.
